# Pathogenicity and enzyme screening of some selected non-dermatophytic moulds

**DOI:** 10.1099/acmi.0.000683.v5

**Published:** 2024-07-08

**Authors:** C.N. Nwofor, N.E. Onyenwe, C.B. Osuoha

**Affiliations:** 1 Department of Microbiology, Imo State University, Owerri, Imo, Nigeria; 2 Department of Pharmaceutical Microbiology, College of Pharmacy, Igbinedion University, Okada, Benin City, Nigeria; 3 Department of Medical Laboratory Science, Imo State University, Owerri, Imo, Nigeria

**Keywords:** animals, hydrolytic enzyme, non-dermatophytes, pathogenicity, virulence

## Abstract

Ten non-dermatophytic moulds isolated from both symptomatic and asymptomatic cattle skin, including *Penicillum citrinum, Aspergillus welwitschiae, Aspergillus aculeatus, Curvularia kusanol, Cladosporium teniussmum, Pestalotiopsis microspora, Fusarium oxysporum, Fusarium linchenicola, Absidia* sp. and *Aspergillus fumigatus,* were subjected to a pathogenicity test using albino mice. These isolates were also screened for five enzymes using a standard plate method. Results from pathogenicity tests showed that *Absidia* sp., *Cladosporium tenuissimum* and *Aspergillus welwitschiae* were able to elicit discoloration, lesion production and alopecia on the albino mice skin, respectively, providing evidence of clinical symptoms associated with cutaneous mycoses. The enzyme screening results revealed the highest zone of activity for keratinase (65 mm), amylase (86 mm), protease (60 mm), lipase (60 mm) and cellulase (86 mm) which were observed on *Pestalotiopsis microspora*, *Aspergillus welwitschiae*, *Cladosporium tenuissimum*, *Aspergillus welwitschiae* and *Aspergillus welwitschiae* respectively. Pathogenicity tests showed that some of these moulds may be virulent and this can be attributed to their possession of some virulence factors, including secretion of hydrolytic enzymes.

## Data Summary

All the supporting data are provided in the paper and Supplementary material.

## Introduction

Dermatophytes are a group of moulds that gain their source of nutrients from non-viable layers of the skin such as hair, nails and feathers and they achieve this by secreting enzymes that hydrolyse them. By this action of hydrolysis, they cause infections called dermatophytosis to these sites of the body [1]. The dermatophytes that affect humans are divided into three genera: *Ttrichophyton*, *Microsporum* and *Epidermophyton* [2]. Dermatophytes are grouped into those that hydrolyse human tissues such as the skin, nails and hair called the anthropophiles, those that dwell in soils and utilize keratin debris present in soils called the geophiles and those that utilize keratin of animal tissues called zoophiles [3]. Due to the wide dissemination of these moulds within the environment, human infections can be caused by both anthropophilic, zoophilic dermatophytes and also by geophilic dermatophytes [1]. Considering the indispensable nature of cattle and the cattle market in Africa, especially in Nigeria, it is imperative to identify the non-dermatophytic moulds that could be responsible for cutaneous mycoses associated with cattle and may be a source of zoonotic infection.

Recently, non-dermatophytic moulds such as *Aspergillus* sp., *Penicillum* sp., *Fusarium* sp. and *Cladosporium* sp., which were normally seen as simply colonizers of these disease surfaces are now observed to be replacing the dermatophytes in causing diseases [4] and this makes diagnosis a challenge [5,6]. Non-dermatophytic moulds have been implicated as causative pathogens in cases of tinea pedis, tinea manuum and onychomycosis [7] and they can also achieve this by secreting enzymes such as keratinase, protease and amylase, which are essential features of fungal pathogenesis [8]. Enzymes are known to play a significant role in infection processes such as nutrition, adhesion, colonization, and penetration of cells and tissues [9]. Dermatophytes have been reported by different studies worldwide as the major causative agents of cutaneous infections [1,3, 10] but there is need to clarify the role of these non-dermatophytic moulds in the infection process. This study subjected some non-dermatophytic moulds to pathogenicity tests using albino mice and also screened them for selected enzymes.

## Methods

### Study area

This study was carried out in the major cattle markets in Abia State and Imo State, southeastern Nigeria.

### Sample collection and microscopy

A survey of the markets was made and each had mature cows for sale and a population of between 50 and 100 cattle. A total of 451 skin scrapings were collected from different parts of the cattle skin with the help of veterinary doctors who were recruited as field assistants. The procedures used were as described previously [11]. Each collected sample was labelled based on location/zone where it was collected, name of the specimen and date of collection. The sample containers were wrapped with aluminium foil and taken to Imo State University microbiology laboratory for analysis. The cattle skin samples were examined by direct microscopy as described previously [12] and then cultured on selected media [11]. Each skin scraping was inoculated and cultured directly on plates of Sabouraud dextrose agar (SDA) supplemented with 20 mg chloramphenicol to inhibit bacteria growth. These were incubated at 37 °C for 1–2 weeks [11]. Discrete pure colonies of each isolate were sub-cultured on SDA slants and were stored in the refrigerator until required for further studies [12] as previously described [11].

### Identification of isolates

The isolates were identified using standard methods [12]. The observations from slide culture (surface and reverse side) of the isolates were compared with a standard mycology atlas for identification [13]. The identification was based on growth rate, colony morphology and microscopy.

### Molecular studies of the isolates

Molecular analysis of the isolates was also carried out as previously described [11]. Extraction was done using a ZR fungal DNA mini prep extraction kit supplied by Inqaba South Africa and extracts were analysed as previously described [11]. The extracted genomic DNA was quantified using a Nanodrop 1000 spectrophotometer [14].

The Internal Transcribed Spacer (ITS) region of the rRNA genes of the fungal isolates were amplified using the primers ITS1F: 5′-CTTGGTCATTTAGAGGAAGTAA-3′ and ITS4: 5′-TCCTCCGCTTATTGATATGC-3′, at the medical laboratory department of Niger Delta University Wilberforce Island, Bayelsa State, Nigeria. Primers on an ABI 9700 Applied Biosystems thermal cycler at a final volume of 40 µl for 35 cycles were used. The PCR mix included: 2× Dream Taq Master mix supplied by Inqaba (Taq polymerase, dNTPs, MgCl), the primers at a concentration of 0.8 µM and the extracted DNA as template. The PCR conditions were as follows: initial denaturation, 95 °C for 5 min; denaturation, 95 °C for 30 s; annealing, 53 °C for 30 s; extension, 72 °C for 30 s for 35 cycles and final extension, 72 °C for 5 min. The product was resolved on a 1 % agarose gel at 130 V for 25 min and visualized on a blue transilluminator [14] as previously described [11].

Gene sequencing was done using the BigDye Terminator kit on a 3510 ABI sequencer by Inqaba Biotechnology as described by Nwofor *et al*. [11]. The sequences obtained were edited using the bioinformatics algorithm Trace edit, and similar sequences were obtained from the National Center for Biotechnology Information (NCBI) database using blastn as described previously [11]. The evolutionary history was inferred using the neighbour-joining method in mega 6.0 [15]. The bootstrap consensus tree inferred from 500 replicates [16] was taken to represent the evolutionary history of the taxa analysed. Evolutionary distances were computed using the Jukes–Cantor method [17] as described by Nwofor *et al*. [11].

### Pathogenecity test of isolates on albino mice

Twenty mice were bought from the Department of Biochemistry of Imo State Polytechnic Umuagwo and examined for skin infection. The mice skin was cleaned with distilled water and mice were fed for 2 weeks before introducing the ten isolates recovered from this study. These mice were paired in twos (male and female) and placed in different cages giving ten different cages. The cages were labelled against the name of the isolate applied. A loopful (fragment) of the isolates was diluted in 5 ml distilled water containing beads, and shaken vigorously to break the fungal strands for homogenization. Tenfold serial dilution was carried out, using 1 ml of the 5 ml stock solution (broken strands) into 9 ml sterile distilled water. For each pair, 0.5 ml of inoculum from 10^−2^ dilution of the isolate was rubbed directly on the mouse at different sites of the skin (e.g neck, waist, leg and viscera) with the aid of a sterile swab stick, and also inoculated into medium containing SDA to verify c.f.u. ml^–1^ of the homogenized fragments used (though results of the c.f.u. ml^–1^ were not included in this paper), while the other pair served as the control [18].

### Enzyme screening test

#### Keratinase screening test

Chicken feathers was precipitated in acetone, allowed to air dry and then ground. This was added to a sterile agar medium as the only source of carbon and nitrogen. The composition of the medium consisted of (g l^−1^): MgCl_2_.6H_2_O (0.1), NaCl (0.5), K_2_HPO_4_ (0.4), KH_2_PO_4_ (0.4), MgCl_2_.6H_2_O (0.1), yeast extract (0.1), chicken feather (10), 1 litre of distilled water and agar (15) (pH 7.5). The isolates were inoculated at the centre of the medium (in duplicate) and then incubated for 6 days at room temperature. Keratinase activity of the fungus was then detected as a clear zone around the colony (in duplicate) and the diameter was measured using a transparent millimetre rule [19] and the mean value was taken.

#### Amylase screening

This was carried out by inoculating the organism individually into PDA medium which was supplemented with 1 g of starch. The agar plates (duplicates) were then incubated at 30 °C for 5 days. After the incubation period, the culture plates were stained with lugols iodine solution and observed for zone of clearance around the colonies. The diameter of hydrolysis formed after the introduction of iodine solution was measured in the two plates to represent the amylolytic activity using a transparent millimetre rule [20] and the mean value was taken.

#### Protease screening test

Protease activity was screened by inoculating isolates on agar medium incorporated with gelatin. The agar medium used was composed of (per litre): 5 g peptone powder, 3 g beef extract, 3 g NaCl, 15 g agar, 1 % gelatin and 1 litre of distilled water (pH 6.0). After incubation at 35 °C for 5–6 days, clear zones around the colony were an indication of protease production and this was observed by flooding with aqueous saturated solution of mercuric chloride reagent (15 g HgCl_2_ dissolved completely in 20 ml of 7 M HCl, then raised to 100 ml with sterile water). The diameter of the zone of clearance in both plates was measured using a transparent millimetre rule [21], and the mean value was taken.

#### Lipase screening test

In this method the medium was prepared using olive oil as the lipidic substrate and phenol red as the indicator. The basal medium was composed of (per litre): 5 g peptone, 2 g yeast extract, 15 g agar, 10 ml olive oil and 10 g phenol red as the indicator. Agar blocks from a 4-day-old culture were inoculated on the basal media plates in duplicate with a control plate (not inoculated). These plates were incubated at 25 °C for 5 days. The presence of lipolysis activity was indicated by yellow coloration around the isolate. The diameter of hydrolysis was measured using a millimetre rule [22], and the mean value was reported.

#### Cellulase screening test

For the cellulose screening test, mineral salt solution, which is composed of (per litre): 0.3. g urea, 1.4 g (NH4)_2_SO_4_, 2.0. g KH_2_PO, 0.3 g CaCl_2_, 0.3 g MgSO_4_, 0.25 g yeast extract and 0.75 g protease peptone, with 10 g l^−1^ carboxymethyl cellulose (CMC) and 17.5 g l^-−^ agar, was prepared [23]. Agar blocks (8 mm in diameter) from 1-week-old fungal colonies were cut and inoculated at the centre of the basal media plates in duplicate with a control. These plates were incubated at 25 °C for 5–7 days. Cellulolytic activity was determined by measuring the diameter of the hydrolysed zone surrounding the colonies after flooding plates with 1 % Congo red dye (0.5–1 h) followed by destaining with 1 M NaCl solution for 15–20 min.

#### Culling of the mice used for pathogenicity testing

All the mice were culled by retrieving them from the cage after 1 month. This was done after monitoring the level at which the fungi were gradually spreading on their skin after the 15th day. Each of the mice was later injected with an anaesthetic agent (pentobarbitone) and immediately transferred into a box with a lid and allowed to die. After confirming death, they were buried appropriately.

## Results and discussion

**Fig. 1.**
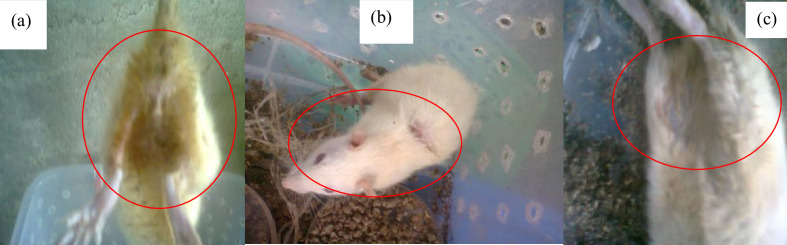
Images showing (**a**) the effects of fungi on albino mice (discoloration) by *Absidia corymbifera*, (**b**) skin lesion on the albino mice by *Cladosporium tenussimum* and (c) loss of hair around the region of the mouse leg (alopecia) by *Aspergillus welwitschiae,* after 10 days of inoculation.

Of the 451 cattle skin scrapings sampled from both states, 25 DNA extracts were sent for purity checking and sequencing. A total of 16 non-dermatophytic moulds were identified phylogenetically (see Supplementary material for Data Summary; Fig. S1, available in the online version of this article) and only ten were selected for the pathogenicity test on the albino mice (also see Supplementary material for Data Summary; Table S1). Of the ten selected non-dermatophytic moulds in this study, only three, *Absidia corymbifera*, *Cladosporium tenuissimum* and *Aspergillus welwitschiae* (Fig. 1), established clinical symptoms suggesting cutaneous mycoses such as discoloration of fur, lesion production and alopecia, respectively, (Fig. 1a–c), while the other isolates, which included *Penicillum citrinum*, *Aspergillus fumigatus*, *Aspergillus aculeatus*, *Fusarium linchenicola*, *Pestalotiopsis microspora*, *Fusarium oxysporium* and *Curvularia kusanol*, did not express any clinical symptoms suggesting cutaneous mycoses within the 1 month of observation.

**Fig. 2.**
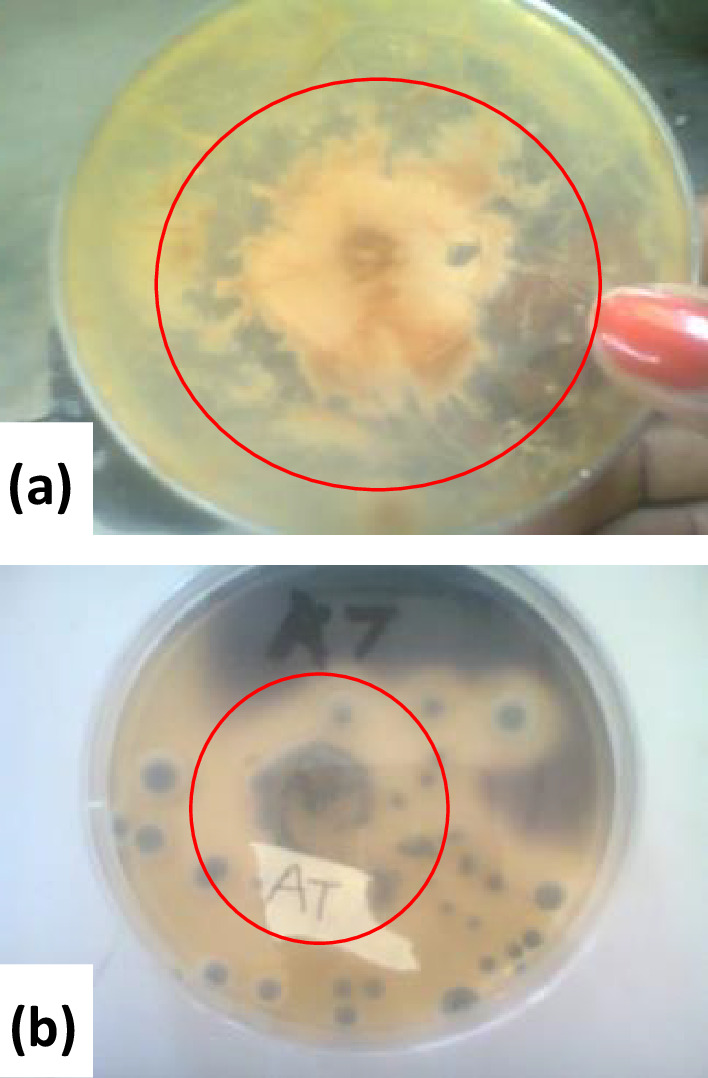
Images of (a) keratin hydrolysis (clear zone on agar plate) by *Curvularia kusanol* and (**b**) zones of starch hydrolysis on agar plate by *Penicillum citrinum*.

The keratinase enzyme screening test was carried out in duplicate (see Table 1), on ten non-dermatophytic moulds, selected for the experiment. Results showed that seven isolates hydrolysed the keratin present while three did not. From the results shown in Table 1, *Penicillum citrinum* grew moderately on the medium and hydrolysed keratin at a diameter of 50 mm*, Aspergillus welwitschiae* grew moderately on the medium and hydrolysed keratin at a diameter of 55 mm*, Aspergillus aculeatus* showed no growth on the medium and no activity was observed, *Curvularia kusanol* grew moderately and showed a hydrolysis diameter at 60 mm (Fig. 2a)*, Cladosporium tenussimum* showed low growth on the medium and hydrolysed keratin at a diameter of 20 mm*, Pestalotiopsis microspora* grew moderately on the medium and showed hydrolysis at 65 mm, *Fusarium oxysporum* grew moderately on the medium and hydrolysed keratin at a diameter of 55 mm, *Fusarium lichenicola* and *Absidia corymbifera* had no growth on the medium and showed no activity, and *Aspergillus fumigatus* showed moderate growth on the medium and keratin hydrolysis at a diameter of 46 mm (Table 1).

**Table 1.** Keratinolytic activity on the non-dermatophytic moulds using chicken feathers

Fungal isolate	Growth on chicken feather agar	Clear zone	Mean diameter of zone (mm)
*Penicillum citrinum*	++	+	50
*Aspergillus welwitschiae*	++	+	55
*Aspergillus aculeatus*	–	–	0
*Curvularia kusanol*	++	+	60
*Cladosporium tenussimum*	+	+	20
*Pestalotiopsis microspora*	++	+	65
*Fusarium oxysporum*	++	+	55
*Fusarium lichenicola*	–	–	0
*Absidia corymbifera*	–	–	0
*Aspergillus fumigatus*	++	+	46

Key: +++, ++, +, – and * represent heavy growth, moderate growth, low growth, no activity and complete hydrolysis respectively.

Amylase screening tests carried out in duplicate on the non-dermatophytic moulds showed *Penicillum citrinum* grew moderately on the medium and hydrolysed the substrate at a diameter of 60 mm (Fig. 2b). *Aspergillus welwitschiae* grew strongly on the medium and hydrolysed starch completely at a diameter of 86 mm, *Aspergillus aculeatus* grew strongly on the medium and hydrolysed starch completely at a diameter of 86 mm, *Curvularia kusanol* showed low growth on the medium and no activity was observed, *Cladosporium tenuissium* grew moderately on the medium and hydrolysed starch with a diameter of 75 mm, *Fusarium oxysporum* grew moderately on the medium and hydrolysed starch at a diameter of 64 mm, *Absidia corymbifera* grew moderately on the medium and hydrolysed starch at 69 mm, *Aspergillus fumigatus* showed slow the growth on medium and hydrolysed starch at a diameter of 30 mm, and *Aspergillus terreus* showed low growth on the medium and hydrolysed starch at a diameter of 37 mm (Table 2).

**Table 2.** Amylase activity on the non-dermatophytic moulds using starch agar plates

Fungal isolate	Growth on starch agar	Clear zone	Mean diameter of zone (mm)
*Penicillum citrinum*	++	+	60
*Aspergillus welwitschiae*	+++	+	86*
*Aspergillus aculeatus*	+++	+	86*
*Curvularia kusanol*	+	−	0
*Cladosporium tenussimum*	++	+	75
*Pestalotiopsis microspora*	−	−	0
*Fusarium oxysporum*	++	+	64
*Fusarium lichenicola*	−	−	0
*Absidia corymbifera*	++	+	69
*Aspergillus fumigatus*	+	+	30

Key: +++, ++, +, − and * represent heavy growth, moderate growth, low growth, no activity and complete hydrolysis respectively.

Proteolytic enzyme activity was determined in duplicate on the ten non-dermatophytic moulds. Seven showed a clear zone of hydrolysis. *Penicillium citrinum* showed slow growth on the medium and hydrolysed substrate at a diameter of 35 mm, *Aspergillus welwitschiae* grew moderately on the medium and hydrolysede substrate at 50 mm, *Aspergillus aculeatus* and *Curvularia kusanol* showed no growth on the medium and no hydrolysis was observed, *Cladosporium tenuissimum* showed heavy growth on the medium and expressed the highest zone of clearance (80 mm) followed by *Fusarium succisae* that grew moderately on the medium but hydrolysed substrate at 60 mm (plate not shown due to poor image resolution), *Absidia corymbifera* grew moderately on the medium and hydrolysed substrate at 50 mm, *Aspergillus terreus* showed slow growth on the medium and hydrolysed substrate at 30 mm, and *Aspergillus flavus* grew moderately on the medium and hydrolysed substrate at 55 mm, while *Fusarium oxysporium* and *Pestalotiopsis microspora* did not grow at all and as such showed no activity on the agar plates (Table 3).

**Table 3.** Proteolytic activity on non-dermatophytic moulds using gelatin agar

Fungal isolate	Growth on gelatin	Clear zone	Mean diameter of zone (mm)
*Penicillum citrinum*	+	+	35
*Aspergillus welwitschiae*	++	+	50
*Aspergillus aculeatus*	−	−	0
*Curvularia kusanol*	−	−	0
*Cladosporium tenussimum*	+++	+	80
*Absidia corymbifera*	++	+	50
*Aspergillus terreus*	+	+	30
*Fusarium succisae*	++	+	60
*Pestalotiopsis microspora*	−	−	0
*Fusarium oxysporum*	−	−	0
*Aspergillus flavus*	++	+	55

Key: +++, ++, +, – and * represent heavy growth, moderate growth, low growth, no activity and complete hydrolysis respectively.

**Fig. 3.**
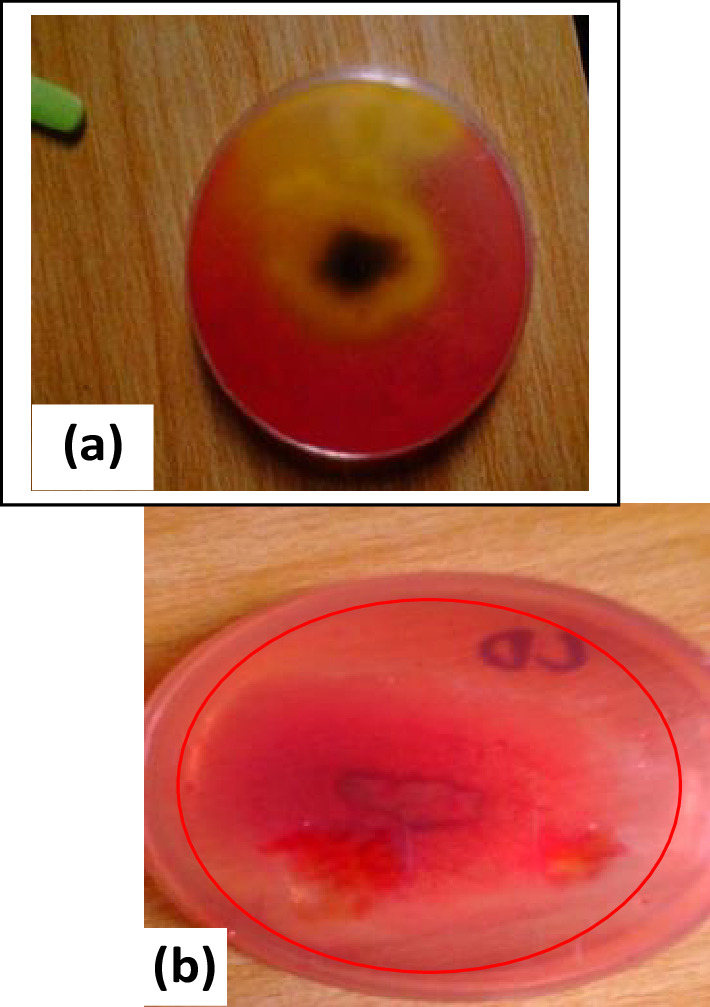
Images of (**a**) hydrolysis of lipid (clear zone on agar plate) by *Aspergillus welwitschiae*, and (**b**) hydrolysis of cellulose on agar plate by *Absidia corymbifera*.

Lipolytic activity was determined in duplicate on the non-dermatophytic moulds and showed that *Penicillum citrinum* had slow growth on the medium, without hydrolysing the substrate. *Aspergillus welwitschiae* grew moderately and hydrolysed the substrate with a diameter of 60 mm (Fig. 3a), *Aspergillus aculeatus* did not grow on the medium and did not hydrolyse the substrate, *Curvularia kusanol* did not grow on the medium and showed no activity, *Cladosporium tenuissium* showed slow growth on the medium with no activity, *Pestalotiopsis microspora* showed no growth on the medium and no activity was observed, *Fusarium lichenicola* grew moderately on the medium and hydrolysed substrate at a diameter of 50 mm, *Absidia corymbifera* showed slow growth on the medium and hydrolysed substrate at a diameter of 35 mm, *Aspergillus fumigatus* did not grow on the medium and no activity was observed, *Aspergillus flavus* grew heavily on the medium and hydrolysed substrate at a diameter of 60 mm while *Fusarium succisae* grew moderately on the medium with a diameter of 50 mm (see Table 4).

**Table 4.** Lipase activity on non-dermatophytic moulds using olive oil with phenol red agar

Fungal isolate	Growth on olive oil with phenol red agar	Clear zone	Mean diameter of zone (mm)
*Penicillum citrinum*	+	−	0
*Aspergillus welwitschiae*	+++	+	60
*Aspergillus aculeatus*	−	−	0
*Curvularia kusanol*	−	−	0
*Cladosporium tenussimum*	+	−	0
*Pestalotiopsis microspora*	−	−	0
*Fusarium lichenicola*	++	+	50
*Absidia corymbifera*	+	+	35
*Aspergillus fumigatus*	−	−	0
*Aspergillus flavus*	+++	+	60
*Fusarium succisae*	++	+	50

Key: +++, ++, +, – and * represent heavy growth, moderate growth, low growth, no activity and complete hydrolysis respectively.

Cellulolytic enzyme activity was determined in duplicate on the non-dermatophytic moulds. Nine showed a zone of hydrolysis. *Penicillum citrinum* showed slow growth on the medium and hydrolysed the substrate at a diameter of 30 mm, *Aspergillus welwitschiae* grew heavily on the medium and hydrolysed the substrate at a diameter of 86 mm*, Aspergillus aculeatus* showed slow growth on the medium and hydrolysed the substrate at a diameter of 35 mm, *Curvularia kusanol* expressed slow growth on the medium and hydrolysed the substrate at a diameter of 30 mm*, Cladosporium tenuissimum* showed low growth on the medium and hydrolysed the substrate at a diameter of 20 mm, *Absidia corymbifera* grew moderately on the medium and hydrolysed the substrate at a diameter of 60 mm (Fig. 3b), *Pestalotiopsis microspore* grew moderately on the medium and hydrolysed the substrate at a diameter of 65 mm*, Fusarium oxysporum* grew moderately on the medium and showed positive cellulolytic activity at a diameter of 65 mm, *Fusarium lichenicola* had slow growth on the medium at a diameter of 10 mm, while *Aspergillus fumigatus* showed no growth on the mediium and showed no signs of cellulolytic activity (Table 5).

**Table 5.** Cellulolytic activity on non-dermatophytic moulds using carboxymethyl cellulose

Fungal isolate	Growth on carboxymethyl cellulose	Clear zone	Mean diameter of zone (mm)
*Penicillum citrinum*	+	+	30
*Aspergillus welwitschiae*	+++	+	86
*Aspergillus aculeatus*	+	+	35
*Curvularia kusanol*	+	+	30
*Cladosporium tenussimum*	+	+	20
*Absidia corymbifera*	++	+	60
*Pestalotiopsis microspora*	++	+	60
*Fusarium oxysporum*	++	+	65
*Aspergillus fumigatus*	−	−	0
*Fusarium lichenicola*	+	+	10

Key: +++, ++, +, – and * represent heavy growth, moderate growth, low growth, no activity and complete hydrolysis respectively.

The above results indicate that some of these non-dermatophytic moulds may be virulent and as such are capable of eliciting superficial infections, as also previously reported [24,25]. A study on *Cladosporium* species revealed that it is capable of infecting animals and humans by causing localized superficial lesions [26]. Another study revealed that *Absidia* species are well-established causative agents of disease in animals and humans [27]. Results for keratinolytic activity by these isolates indicate that they can hydrolyse keratinous substances, some of the hardest substrates to degrade, and thus can justify the reason why some of these non-dermatophytic moulds were able to elicit clinical symptoms on albino mice skin [28,30]. Results from aminolytic activity from our study showed that *Aspergillus* species and *Fusarium* species are good producers of amylase [31,32].

## Conclusion

1. The ability to secrete protease enzymes has been related to fungal pathogenicity [33]. This certainly justifies the ability of *Cladosporium tenuissimum* to elicit lesions on albino mice in our study [34,37]. The inability of some non-dermatophytic moulds to grow on the media could be due to their genetic constitution, or they did not find the environment to be enabling and thus could not express their genetic trait. For cellulolytic screening all isolates showed zones of clearance except *Aspergillus fumigatus*. This result agrees with findings from other studies [32,38, 39].
2. Our results from the lipolytic test were in agreement with previous studies [32,38, 39]. The skin possesses sebaceous glands that secret lipids which can be hydrolysed by lipase. This could explain why *Aspergillus welwitschiae* was able to produce alopecia on the albino mice skin. This finding agrees with other studies where *Aspergillus* spp. were demonstrated as lipase-producing fungi on palm oil mill effluent [40,41]. Another study demonstrated *Cladosporium langeronii* as a good lipase producer [42] which was not in line with our study.
3. However, infections caused by these organisms could reduce the economic value of the cattle and the cattle market. Thus, in the treatment of these infections, antifungal therapy is usually employed, but overuse or misuse reduce the efficacy of the treatment of this disease. Alternative remedies such as medicinal plants could be used. One of the plants suggested to be used based on our previous analysis is *Mitracarpus scaber,* which has been found to possess some antifungal properties [43].
4. Regular checks by veterinary doctors should be ensured and during transit cattle should be kept on columns in trucks to reduce skin trauma and avoid opportunistic infections due to non-dermatophytes [44].

## supplementary material

10.1099/acmi.0.000683.v5 Uncited Supplementary Material 1.

## Abbreviations

ITS: internal transcribed spacer
SDA: sabouraud dextrose agar
