# Supplementary material for: Pathogenicity and enzyme screening of some selected non-dermatophytic moulds
Source: Access Microbiol. 2024 Jul 8;6(7):000683.v5. doi: 10.1099/acmi.0.000683.v5 (PMC11316586; doi:10.1099/acmi.0.000683.v5)
Supplement: Uncited Supplementary Material 1. [file acmi-6-00683-s001.pdf]

Supplementary material for Data Summary

Table 1: Shows the report of the nucleic acid analysis for 10 isolate used for Pathogenicity Test

Sample ID	ng/ul	A260	A280	260/280	260/230	340 raw
Penicillum citrinum	33.38	0.668	0.377	1.77	0.49	0.124
Aspergillus welwitschiae	75.26	1.505	0.810	1.86	0.30	-0.027
Aspergillus aculeatus	95.25	1.905	0.991	1.92	1.54	0.025
Curvularia kusanol	40.94	0.819	0.421	1.94	0.42	0.022
Cladosporium tenussimum	22.32	0.446	0.222	2.01	0.21	0.031
Pestalotiopsis microspora	51.57	1.031	0.549	1.88	0.42	-0.026
Fusarium lichenicola	2.78	0.056	0.028	1.98	0.09	0.022
Absidia sp.	2.32	0.046	0.011	4.32	0.05	0.020
Aspergillus fumigatus	3.58	0.072	0.039	1.83	0.04	0.028
Fusarium oxysporum	43.20	0.864	0.564	1.53	0.37	0.924

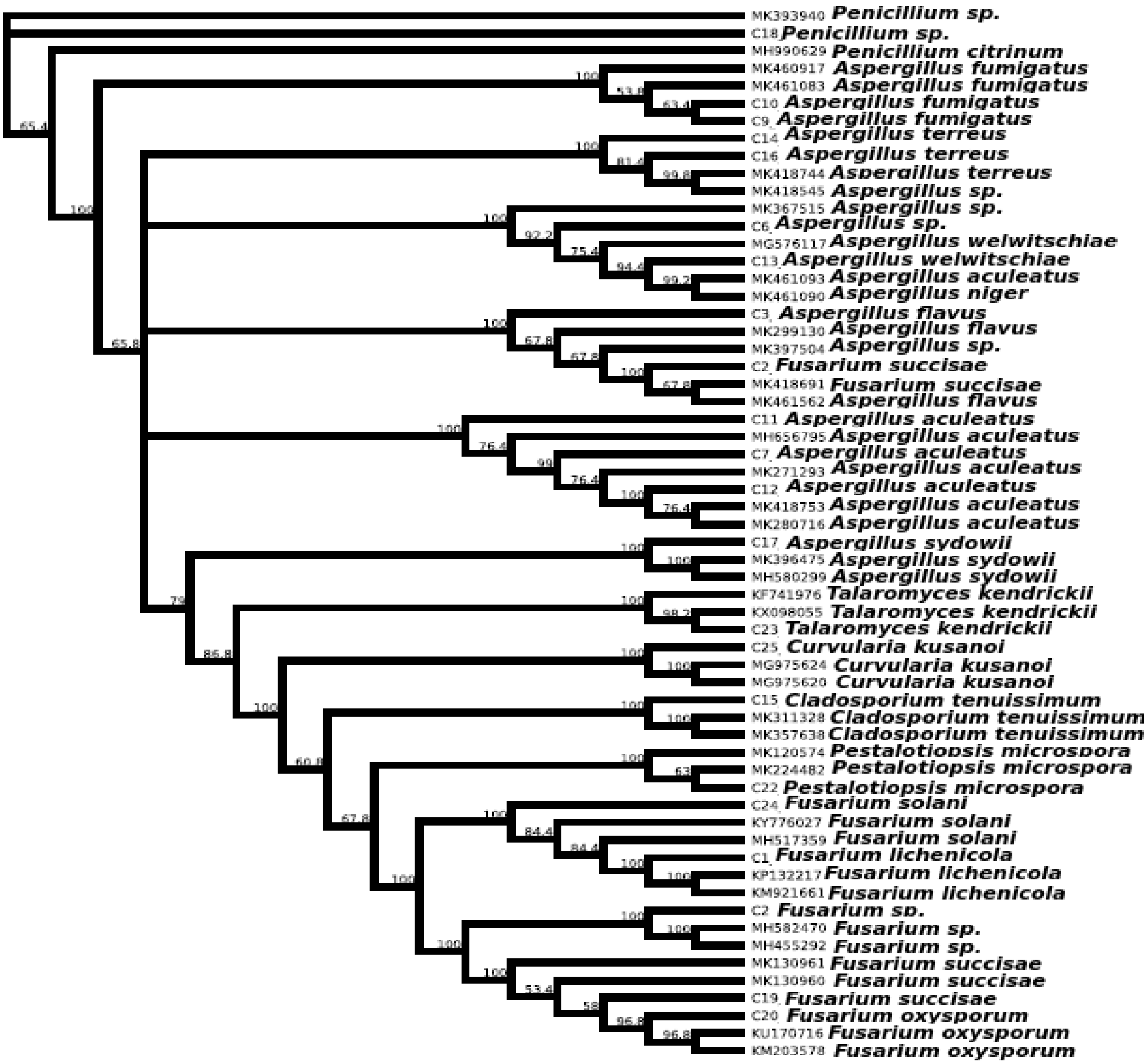

Figure 1: The phylogenetic tree of the result from the internal transcriber spacer (ITS) obtained from the isolate from this study
